# Violations of the right to food during deprivation of liberty: a global socio-legal assessment of United Nations Human Rights Treaty Bodies and the European Committee for the Prevention of Torture and Inhuman or Degrading Treatment or Punishment mission reporting on selected prisons since 2015

**DOI:** 10.1186/s12889-025-25310-7

**Published:** 2025-11-06

**Authors:** Marie Claire Van Hout, Ulla-Britt Klankwarth, Lisa Glaum, Heino Stöver

**Affiliations:** 1 South East Technological University, Cork Road Campus, Cork Road, Waterford, X91 K0EK Ireland; 2 Institute for Addiction Research, Faculty of Health and Social Work, Frankfurt University of Applied Sciences, Nibelungenplatz 1, D- 60318 Frankfurt am Main, Germany; 3 Faculty of Public Health, Bielefeld University, Universitätsstraße 25, D-33615 Bielefeld, Germany

**Keywords:** Food, Nutrition, Hunger, Prison, Deprivation of liberty, Human rights

## Abstract

**Background:**

States have a heightened duty of care owed to persons deprived of their liberty extending beyond the prohibition of torture and discrimination. Due to their complete reliance on the State, provision of adequate and quality nutrition in prison is a fundamental human right of those detained. Failure to meet the basic requirements of sustenance or deny/restrict food constitutes cruel, inhuman or degrading treatment, or even torture.

**Methods:**

In order to examine global progress in protecting and upholding the rights of people living in prisons to adequate food and nutrition, we conducted a global socio-legal assessment of the United Nations (UN) Human Rights Treaty Bodies (Committee against Torture, Committee for the Rights of the Child, Human Rights Committee, Committee on the Elimination of Discrimination against Women); and the European Committee for the Prevention of Torture and Inhuman or Degrading Treatment or Punishment (CPT) mission reporting on selected prisons since 2015. A comprehensive global search in English and French was conducted on the Council of Europe and the UN Human Rights Treaty databases. Following double screening, the final dataset of 237 reports spanning six continents (129 countries) was charted, tabularized against norms and standards (UN Nelson Mandela Rules, Bangkok Rules, the European Prison Rules) and analyzed thematically.

**Results:**

Identified areas of concern and possible human rights violations documented by prison inspections centered on six key themes: *geographies where the right to adequate food in prisons is of concern; inadequacy of food provision; poor food preparation practices*,* environmental health standards and disease; reliance on external support for food*,* corruption and exploitation; food as punishment and control measure;* and *vulnerability of special populations in prison.*

**Conclusions:**

Despite international and regional human rights norms and assurances, prison inspections revealed that standards and adequacy of food and nutrition in prisons are often lacking due to resource scarcity, violence, punishment, inter-personal dynamics and corruption. UN Human Rights Treaty Bodies and CPT inspections must continue to thoroughly assess food standards and provision in prisons, ensure that the denial or restriction of food as punishment is prohibited, and include a focus on those with gender and age-related, religious and medical needs.

**Supplementary Information:**

The online version contains supplementary material available at 10.1186/s12889-025-25310-7.

## Background

The global prison population of 11.5 million on any given day continues to rise, with an increase of 24% observed since 2000 [1]. The number of people who cycle in and out of prison each year however remains undetermined. One in three individuals in prison globally are presumed innocent and held on remand [1]. The poor, marginalized, members of ethnic minority and Indigenous peoples, and key populations (gay men and other men who have sex with men, sex workers, transgender people, people who inject drugs) are disproportionately affected by incarceration [1]. – [2] Prison overcrowding remains a substantial issue globally, generally due to excessive use of pre-trial detention, with 61% of countries reported to be operating above capacity, 34% between 100 and 150%, and 27% above 150% [3]. – [4] Overcrowding substantially affects the availability of resources including access to clean drinking water, food, sanitation and hygiene, and appropriate healthcare [1]. Due to their complete reliance on the state for protection and basic needs provisions, people living in prison are vulnerable to the consequences of spatial and resource scarcity, which can include corruption, inter-personal violence, predatory behaviors and transactional sexual activity in return for protection or privilege (basic provisions including soap, bedding and food) [1, 3, 5–8]. The 2024 report by the United Nations (UN) Special Rapporteur on Torture and other cruel, inhuman or degrading treatment or punishment recognized the ongoing challenge of prison overcrowding by stating *“(.) the struggle for space and resources within prisons create circumstances in which torture*,* violence*,* and corruption thrive.”* [8].

## Reliance on the state and basic human right to food

States have a heightened duty of care owed to persons deprived of their liberty extending beyond the prohibition of torture and discrimination [8] and are obliged to ensure that sufficient and adequate food is available to all detainees [9]. A special diet must be provided to certain detainee categories (pregnant women, breastfeeding women, children, people with certain medical needs) and in accordance with cultural and religious practices [9]. The UN Human Rights Committee outlines that *“(.) the State party by arresting and detaining individuals takes the responsibility to care for their life”*, and that it is *“incumbent on States to ensure the right of life of detainees*,* and not incumbent on the latter to request protection.”* [10] These responsibilities are relevant to all aspects of the right to life, health and well-being, and protection from harm.

Rule 1 of the UN Standard Minimum Rules for the Treatment of Prisoners (the Nelson Mandela Rules) states that *“[a]ll prisoners shall be treated with the respect due to their inherent dignity and value as human beings. No prisoner shall be subjected to*,* and all prisoners shall be protected from*,* torture and other cruel*,* inhuman or degrading treatment or punishment.”* [11] The UN Special Rapporteur on Torture has emphasized that *“treatment and conditions within prison are not intended to be an additional hardship or penalization; and must never be degrading*,* inhuman or cruel.”* [8] Failure to meet the basic requirements of sustenance or deny/restrict food constitutes cruel, inhuman or degrading treatment, or even torture [9]. Deprivation of food and water is prohibited in all circumstances, including as a form of disciplinary sanction [9, 11]. 

The Council of Europe’s (CoE) European Prison Rules and the European Committee for the Prevention of Torture and Inhuman or Degrading Treatment or Punishment (CPT) publish prison standards which include a focus on food provision, nutrition and medical oversight of food quality and amount in European prisons [12, 13]. The CPT engages in routine monitoring of prisons by assessing how prisoners are treated and has been explicit that *“(.)every effort should be made to meet the specific dietary needs of pregnant women prisoners*,* who should be offered a high protein diet*,* rich in fresh fruit and vegetables”* (Paragraph 26) [14]. In 2012, the CPT recognized the cultural needs of foreign prisoners detained in Europe by stating that *“in addition to providing a nutritious diet that takes account of the cultural and religious requirements of prisoners*,* prison authorities shall*,* where possible*,* provide prisoners with opportunities to purchase and cook food that makes their diet more culturally appropriate and to take their meals at times that meet their religious requirements.”* [15] The CPT has also underscored the role of healthcare staff in monitoring standards of food adequacy and nutrition, and reiterated that *“health-care staff should also play an active role in monitoring the quality and quantity of food. The juveniles’ nutritional state should be assessed through*,* inter alia*,* drawing up a growth chart for those juveniles who are still in the growth phase”* (Paragraph 118) [16]. See Table 1.

**Table 1:** Normative standards of detention relevant to food and nutrition in prison

United Nations Standard Minimum Rules for the Treatment of Prisoners (the Nelson Mandela Rules)
Rule 22.1 *Every prisoner shall be provided by the prison administration at the usual hours with food of nutritional value adequate for health and strength*,* of wholesome quality and well prepared and served.* Rule 35.1 *The physician or competent public health body shall regularly inspect and advise the prison director on:* * (a) The quantity*,* quality*,* preparation and service of food […]* Rule 42 *General living conditions addressed in these rules*,* including those related to light*,* ventilation*,* temperature*,* sanitation*,* nutrition*,* drinking water*,* access to open air and physical exercise*,* personal hygiene*,* health care and adequate personal space*,* shall apply to all prisoners without exception.* Rule 43.1 *In no circumstances may restrictions or disciplinary sanctions amount to torture or other cruel*,* inhuman or degrading treatment or punishment. The following practices*,* in particular*,* shall be prohibited*: * (a) Indefinite solitary confinement;* * (b) Prolonged solitary confinement;* * (c) Placement of a prisoner in a dark or constantly lit cell;* * (d) Corporal punishment or the reduction of a prisoner’s diet or drinking water;* * (e) Collective punishment*
United Nations Rules for the Treatment of Women Prisoners and Non-Custodial Measures for Women Offenders (the Bangkok Rules)
Rule 48.1 *Pregnant or breastfeeding women prisoners shall receive advice on their health and diet under a programme to be drawn up and monitored by qualified health practitioner. Adequate and timely food*,* a healthy environment and regular exercise opportunities shall be provided free of charge for pregnant women*,* babies*,* children and breastfeeding mothers.* Rule 48.2 *Women prisoners shall not be discouraged from breastfeeding their children*,* unless there are specific health reasons to do so.* Rule 48.3 *The medical and nutritional needs of women prisoners who have recently given birth*,* but whose babies are not with them in prison*,* shall be included in treatment programmes.*
United Nations Rules for the Protection of Juveniles Deprived of their Liberty
Rule 37 *Every detention facility shall ensure that every juvenile receives food that is suitably prepared and presented at normal meal times and of a quality and quantity to satisfy the standards of dietetics*,* hygiene and health and*,* as far as possible*,* religious and cultural requirements.*
Council of Europe European Prison Rules
Rule 22.1 *Prisoners shall be provided with a nutritious diet that takes into account their age*,* health*,* physical condition*,* religion*,* culture and the nature of their work.* Rule 22.2 *The requirements of a nutritious diet*,* including its minimum energy and protein content*,* shall be prescribed in national law*. Rule 22.3 *Food shall be prepared and served hygienically.* Rule 22.4 *There shall be three meals a day with reasonable intervals between them.* Rule 22.6 *The medical practitioner or a qualified nurse shall order a change in diet for a particular prisoner when it is needed on medical grounds.*
Principles and Best Practices on the Protection of Persons Deprived of Liberty in the Americas
Principle XI − 1. Food *Persons deprived of liberty shall have the right to food in such a quantity*,* quality*,* and hygienic condition so as to ensure adequate and sufficient nutrition*,* with due consideration to their cultural and religious concerns*,* as well as to any special needs or diet determined by medical criteria. Such food shall be provided at regular intervals*,* and its suspension or restriction as a disciplinary measure shall be prohibited by law.*

### Food for thought: rationale for the study

Despite international and regional human rights assurances regarding access to adequate nutrition in prisons, and that prison food represents a unique opportunity to enhance the health and wellbeing of a very marginalized often chronically unwell population, standards of adequate nutrition are often lacking, and heavily dependent on budgetary and security issues [17]. Various studies have reported on assessments of nutritional and caloric content of food in prisons, both in developed and low resource settings [18–27]. Food insecurity, and food deprivation due to poor prison conditions or as instrument of torture have been documented in the United States, the Russian Federation, Italy, Zimbabwe, Sudan, Mozambique, Gabon, Palestine, Tanzania, Ethiopia, Côte d’Ivoire, Democratic Republic of Congo, Kenya, Malawi, Haiti and Ghana.[9, 22–24, 28–31] Furthermore, reports from multiple countries highlight instances of malnutrition related deaths among people living in prison [30, 32–34]. 

There are several comprehensive systematic and scoping reviews on food in prisons [20]. – [21, 35–37] They along with various sociological studies illuminate how food is a central commodity in prison life and how food plays a substantial role in shaping the carceral experience [37–40]. The symbolism and significance of food within daily life in confinement are particularly central to the construction of prisoner identity and status, the underground economy of food transactioning and intimidation and are a means through which institutional and social contempt for people in prison is communicated to and embodied by the prison community [41–45]. An enhanced prisoner status can counter notions of food as threat and poison, through systems of bartering, solidarity and recompense [45]. Prison food has multiple meanings within the carceral experience, relating to food relationships underpinned by the appreciation of food itself (taste, variety and nutritional content), hunger, unwanted bodily changes (weight loss, gain), fear of food due to official mistrust, the dynamics of institutional power and punishment, hunger strike as a form of political statement, and the dynamics relating to food preparation, timing of meals, sharing and consumption of food [39, 46, 47] Prison foodways^1^ are also key to unequal power dynamics, social networks and relationships, violence, and the manipulation and abusive use of food supply as control measure by authorities [36, 39, 46, 48, 49] The complex symbolism of food in women’s prisons is threefold; as a gendered locus of power and resistance, as a conduit to family relationships and as a reflection of lived experience in prison [50–55].

In order to examine global progress in protecting and upholding the rights of people living in prisons to adequate food and nutrition, we conducted a global socio-legal assessment of UN Human Rights Treaty Bodies (Committee against Torture (CAT), Committee for the Rights of the Child (CRC), Human Rights Committee, Committee on the Elimination of Discrimination against Women (CEDAW)); and the CPT mission reporting on violations of norms and standards relating to adequate food and nutrition in prisons since 2015. Recommended questions regarding food for prison monitors from the Association for the Prevention of Torture [56] are presented in Table 2 [56].

**Table 2:** Recommended questions regarding Food for Prison Monitors [56]

Does domestic law stipulate quality criteria for meals served to detainees? What is the annual food budget of the prison per detainee? At what time and how are meals served? How much time are detainees allowed to eat their meals? How is the menu selected? Are health personnel involved in the menu selection process? Do the detainees have the opportunity to submit suggestions regarding their meals? Are inspections concerning hygiene and the nutritional value of the meals carried out regularly? What are the current meal arrangements for detainees during their transfer or for persons who have just arrived in the institution? Where is the kitchen located, and what are its conditions of hygiene and ventilation? Are food warehouses protected against moisture and other harmful elements? Are food stocks, including product expiration dates, checked regularly? Are the detainees allowed to receive food from their families? Are detainees and their families familiar with the restrictions in this regard? Are precautions taken to avoid fire or electrocution when detainees cook meals in their cells? What products are available in the prison shop? Are their prices affordable/equivalent to those of products sold outside? Is there any indication that food management is controlled by certain categories of detainees? If so, what is the impact on the most vulnerable detainees and what remedial measures are taken by the authorities? Do disciplinary sanctions involve the deprivation or restriction of water and/or food? In cases where the meal and/or shop services are outsourced to a private company does this have any impact, either positive or negative, on the prison population? *Minorities and indigenous people* Are the meals served to detainees from ethnic, indigenous or religious minority groups adapted to their customs and beliefs? Do detainees from ethnic, indigenous or religious minority groups have the right to observe fasting periods in accordance with their religion or beliefs? If necessary, are arrangements to accommodate fasting periods put in place by the authorities? Can detainees interrupt their fast at any time? *Women* Do pregnant or breastfeeding inmates receive appropriate food? Do they receive the necessary support of qualified health personnel? Do detainees who have recently given birth but whose children are not with them in prison receive a diet tailored to their specific nutritional needs? *Children* Do detained minors benefit from adequate nutrition adapted to their growth?	

## Methodology

The assessment sits squarely within a socio-legal theoretical approach by considering the social and health related impacts of the law *‘in action’* on the degree to which right to adequate food and nutrition during deprivation of liberty is upheld in prisons, as reported by the UN Human Rights Treaty Bodies and the CPT when inspecting standards. A comprehensive global search restricted to 2015–2025 was conducted on the Council of Europe and the United Nations Human Rights treaty databases. *Concluding Observations* promulgated from the Human Rights Committee, the CAT, the CRC and the CEDAW, and the ad-hoc and periodic mission reports of the European CPT were carefully screened in chronological order using English and French search terms. See Table 3.

**Table 3:** UN Human Rrights Treaty Bodies and European CPT reporting on the right to adequate food and nutrition in prisons (2015–2025)

Treaty Body	Total Concluding Observations, 2015–2025	Search Terms	Final Data Set
CAT	146	English: food; nutri*tion*tional*tious*tionist; cook*ing; diet*ician*ary; meal; kitchen*ette French: nour*riture; alim*ent; nutri*tion; cuisin*er; mange*r; diète; repas	65
Human Rights Committee	163		42
CEDAW	222		6
CRC	182		25
Council of Europe	Total CPT Reports, 2015–2025	Search Terms	Final Data Set
CPT	131 (75 periodic, 56 ad hoc)	English: food; nutri*tion*tional*tious*tionist; cook*ing; diet*ician*ary; meal; kitchen*ette French: nour*riture; alim*ent; nutri*tion; cuisin*er; mange*r; diète; repas	99 (69 periodic, 30 ad hoc)
Total Records	844		237

Each report was carefully examined, by scrutinizing the extant reference to standards of detention, particularly focused on right to adequate food and nutrition in prison in that country. French language reports were translated into English by the second author using computer assisted translation and checked by the last author (fluent). Following double screening by the second and third authors, the final data set (*n* = 237 records) was cross-checked by the first author.

The final data set was charted [see supplementary online material] and documented areas of Committee concern pertaining to a country’s provision of food to people in prison, including where multiple recommendations were made to improve the standard of food in prisons (with insufficient improvement), and where violations of the right to food were identified within the parameters of adequate standards of detention as provided by the UN and European normative standards of care in prisons (see Tables 1, 4 and 5).

**Fig. 1:**
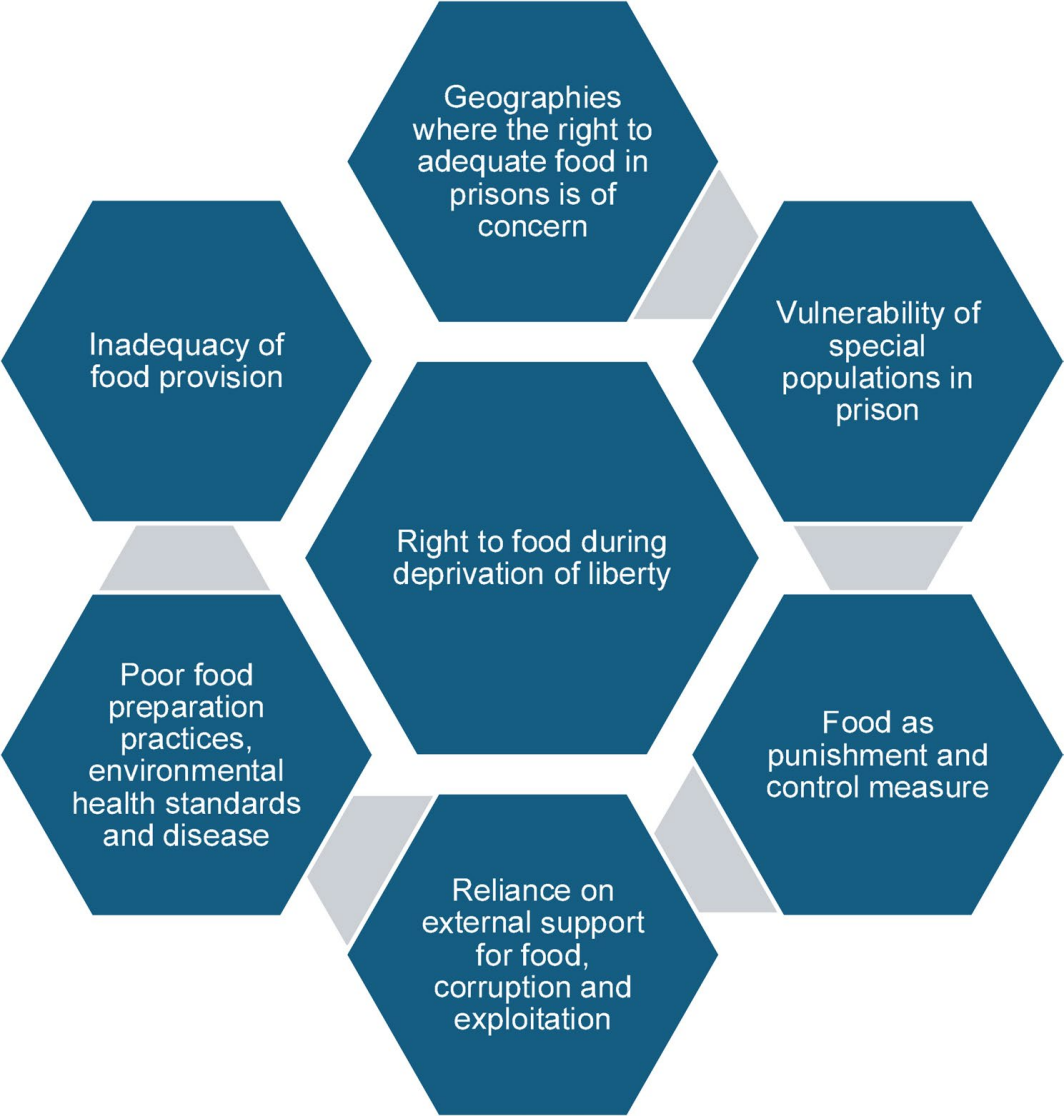
Themes

Thematic analysis of inspection narratives was also undertaken with six emerging key themes: *geographies where the right to adequate food in prisons is of concern; inadequacy of food provision; poor food preparation practices*,* environmental health standards and disease; reliance on external support for food*,* corruption and exploitation; food as punishment and control measure;* and *vulnerability of special populations in prison.* See Fig. 1.

## Results

### Geographies where the right to adequate food in prisons is of concern

The final dataset of 237 reports reflected 129 countries, with the geographical scope spanning six continents. Countries were concentrated in Africa, Europe and Asia, with smaller numbers in the Americas and Australia. The distribution of reports across countries varied, with repeat mission reports in Lithuania, the Republic of Moldova, and the United Kingdom (*n* = 6); Bulgaria (*n* = 5); and Armenia, Belgium, Bosnia and Herzegovina, Greece, North Macedonia, and Ukraine (*n* = 4). A further 16 countries had three reports each, and 39 countries had two reports. The remaining countries had a single report each (*n* = 64). See Table 4.

**Table 4:** Geographies of identified UN Human Rights Treaty Bodies and European CPT concerns regarding violation of the right to adequate food in prisons worldwide

Continent	Total reports (*n* = 237)	Total countries (*n* = 129)	Countries and number of reports per country
Africa	*n* = 65	*n* = 40	Angola (*n* = 2), Benin (*n* = 2), Botswana (*n* = 1), Burkina Faso (*n* = 3), Burundi (*n* = 3), Cabo Verde (*n* = 1), Cambodia (*n* = 1), Cameroon (*n* = 1), Central African Republic (*n* = 1), Chad (*n* = 1), Côte d’Ivoire (*n* = 2), Democratic Republic of the Congo (*n* = 2), Djibouti (*n* = 1), Egypt (*n* = 1), Equatorial Guinea (*n* = 1), Eritrea (*n* = 2), Ethiopia (*n* = 2), Gambia (*n* = 1), Guinea (*n* = 2), Kenya (*n* = 1), Kiribati (*n* = 1), Lesotho (*n* = 2), Liberia (*n* = 1), Madagascar (*n* = 2), Malawi (*n* = 2), Mauritania (*n* = 1), Mauritius (*n* = 2), Namibia (*n* = 3), Niger (*n* = 3), Nigeria (*n* = 2), Rwanda (*n* = 2), Senegal (*n* = 1), Seychelles (*n* = 1), Somalia (*n* = 2), South Africa (*n* = 2), Togo (*n* = 2), Tunisia (*n* = 1), Uganda (*n* = 1), Zambia (*n* = 2), Zimbabwe (*n* = 1)
Asia	*n* = 42	*n* = 31	Afghanistan (*n* = 1), Armenia (*n* = 4), Azerbaijan (*n* = 3), Bahrain (*n* = 1), Bangladesh (*n* = 1), Cyprus (*n* = 2), Georgia (*n* = 2), India (*n* = 1), Indonesia (*n* = 1), Iran (*n* = 1), Iraq (*n* = 1), Israel (*n* = 1), Jordan (*n* = 2), Kuwait (*n* = 1), Kyrgyzstan (*n* = 1), Lao People’s Democratic Republic (*n* = 1), Lebanon (*n* = 1), Maldives (*n* = 1), Pakistan (*n* = 1), Palestine (*n* = 1), Philippines (*n* = 2), Qatar (*n* = 1), Republic of Korea (*n* = 1), Saudi Arabia (*n* = 1), Syrian Arab Republic (*n* = 1), Tajikistan (*n* = 1), Thailand (*n* = 1), Turkmenistan (*n* = 2), Uzbekistan (*n* = 1), Viet Nam (*n* = 2), Yemen (*n* = 1)
Australia	*n* = 2	*n* = 2	Samoa (*n* = 1), Solomon Island (*n* = 1)
Europe	*n* = 104	*n* = 42	Albania (*n* = 3), Andorra (*n* = 1), Austria (*n* = 1), Belgium (*n* = 4), Bosnia and Herzegovina (*n* = 4), Bulgaria (*n* = 5), Croatia (*n* = 2), Czech Republic (*n* = 1), Denmark (*n* = 1), Estonia (*n* = 1), Finland (*n* = 1), France (*n* = 2), Germany (*n* = 2), Greece (*n* = 4), Hungary (*n* = 2), Iceland (*n* = 1), Ireland (*n* = 2), Italy (*n* = 3), Kosovo (*n* = 2), Latvia (*n* = 2), Liechtenstein (*n* = 1), Lithuania (*n* = 6), Luxembourg (*n* = 2), Malta (*n* = 1), Monaco (*n* = 1), Montenegro (*n* = 2), North Macedonia (*n* = 4), Poland (*n* = 2), Portugal (*n* = 3), Republic of Moldova (*n* = 6), Romania (*n* = 2), Serbia (*n* = 3), Slovak Republic (*n* = 1), Slovenia (*n* = 1), Spain (*n* = 3), Sweden (*n* = 2), Switzerland (*n* = 3), The Netherlands (*n* = 3), Ukraine (*n* = 4), United Kingdom (*n* = 6), Russian Federation (*n* = 1), Türkiye (*n* = 3)
North America	*n* = 12	*n* = 8	Antigua and Barbuda (*n* = 1), Belize (*n* = 1), Canada (*n* = 1), Cuba (*n* = 1), Guatemala (*n* = 1), Honduras (*n* = 2), Mexico (*n* = 2), Nicaragua (*n* = 3)
South America	*n* = 12	*n* = 6	Argentina (*n* = 3), Brazil (*n* = 2), Ecuador (*n* = 2), Peru (*n* = 3), Suriname (*n* = 1), Venezuela (*n* = 1)

Table 5 illustrates the identified areas of concern and violations of UN and CoE norms and standards relating to standards of detention. See Table 5.

**Table 5:** Identified violations of rules explicit to food and nutrition in prison

United Nations Standard Minimum Rules for the Treatment of Prisoners (the Nelson Mandela Rules)	Treaty Body report and Countries
Rule 22.1 *Every prisoner shall be provided by the prison administration at the usual hours with food of nutritional value adequate for health and strength*,* of wholesome quality and well prepared and served.*	Not explicitly referred to in Treaty Body reports.
Rule 42 *General living conditions addressed in these rules*,* including those related to light*,* ventilation*,* temperature*,* sanitation*,* nutrition*,* drinking water*,* access to open air and physical exercise*,* personal hygiene*,* health care and adequate personal space*,* shall apply to all prisoners without exception.*	CAT (Afghanistan, 2017; Argentina, 2017; Armenia, 2017; Bahrain, 2017; Bangladesh, 2019; Belgium, 2021; Benin, 2019; Brazil, 2023; Bulgaria, 2017; Burkina Faso, 2019; Burundi, 2023; Cameroon, 2024; Canada, 2018; Chad, 2022; Côte d’Ivoire, 2024; Cuba, 2022; Democratic Republic of the Congo, 2019; Ecuador, 2024; Egypt, 2023; Ethiopia, 2023; Guatemala, 2018; Honduras, 2024; Iraq, 2022; Jordan, 2016, 2024; Kenya, 2022; Kiribati, 2023; Lebanon, 2017; Lithuania, 2021; Malawi, 2022; Maldives, 2018; Mauritania, 2018; Mauritius; 2017; Namibia, 2017, 2024; Nicaragua, 2022, 2022; Niger, 2019; Nigeria, 2021; Palestine, 2022; Peru, 2018; Philippines, 2016; Poland, 2019; Qatar, 2019; Russian Federation, 2018; Rwanda, 2017; Senegal, 2019; Somalia, 2022; South Africa, 2019; Tajikistan, 2018; Tunisia, 2016; Turkmenistan, 2017; Viet Nam, 2018) Human Rights Committee (Angola, 2019; Belize, 2018; Benin, 2015, Brazil, 2023, Burkina Faso, 2016; Burundi, 2023, Côte d’Ivoire, 2015; Democratic Republic of the Congo, 2017; Ecuador, 2024; Equatorial Guinea, 2019; Eritrea, 2019; Ethiopia, 2022; Gambia, 2018; Greece, 2024; Guinea, 2018; India, 2024; Iran, 2023; Lao People’s Democratic Republic, 2018; Lesotho, 2023; Liberia, 2018; Lithuania, 2018; Madagascar, 2017; Namibia, 2024; Nicaragua, 2022; Niger, 2019; Nigeria, 2019; Pakistan, 2024; Peru, 2023; Philippines, 2022; Somalia, 2024; South Africa, 2016; Suriname, 2024; Syrian Arab Republic, 2024; Thailand, 2017; Türkiye, 2024; Turkmenistan, 2017; Uganda, 2023; Uzbekistan, 2020; Venezuela, 2023; Zambia, 2023) CEDAW (Burundi, 2016; Central African Republic, 2024; Burkina Faso, 2017; Niger, 2017)
Rule 35.1 *The physician or competent public health body shall regularly inspect and advise the prison director on: (a) The quantity*,* quality*,* preparation and service of food […].*	Not explicitly referred to in Treaty Body reports.
Rule 43.1 *In no circumstances may restrictions or disciplinary sanctions amount to torture or other cruel*,* inhuman or degrading treatment or punishment. The following practices*,* in particular*,* shall be prohibited*: *(d) Corporal punishment or the reduction of a prisoner’s diet or drinking water;*	CAT (Botswana, 2022; Kuwait, 2024; Viet Nam, 2018)
United Nations Rules for the Treatment of Women Prisoners and Non-Custodial Measures for Women Offenders (Bangkok Rules)	
Rule 48.1 *Pregnant or breastfeeding women prisoners shall receive advice on their health and diet under a programme to be drawn up and monitored by qualified health practitioner. Adequate and timely food*,* a healthy environment and regular exercise opportunities shall be provided free of charge for pregnant women*,* babies*,* children and breastfeeding mothers.*	CEDAW (Honduras, 2022; Yemen, 2021) CRC (Bulgaria, 2016; Eritrea, 2015; Zimbabwe, 2016)
Rule 48.3 *The medical and nutritional needs of women prisoners who have recently given birth*,* but whose babies are not with them in prison*,* shall be included in treatment programmes.*	CAT (Türkiye, 2024)
United Nations Rules for the Protection of Juveniles Deprived of their Liberty	
Rule 37 *Every detention facility shall ensure that every juvenile receives food that is suitably prepared and presented at normal meal times and of a quality and quantity to satisfy the standards of dietetics*,* hygiene and health and*,* as far as possible*,* religious and cultural requirements.*	CRC (Argentina, 2024; Bulgaria, 2016, Guinea, 2019; Israel, 2024; Republic of Korea, 2019; Samoa, 2016; Solomon Islands, 2018)
Council of Europe European Prison Rules	CPT report and Countries
Rule 22.1 *Prisoners shall be provided with a nutritious diet that takes into account their age*,* health*,* physical condition*,* religion*,* culture and the nature of their work.*	Armenia, 2021; Belgium, 2018; Bulgaria, 2018, 2022; Denmark, 2024; Estonia, 2024; Greece, 2022; Hungary, 2024; Ireland, 2020; Italy, 2017, 2020, 2023; Lithuania, 2018, 2019, 2023, 2024; Malta, 2016; Montenegro, 2023; North Macedonia, 2017, 2021, 2021, 2024; Portugal, 2018, 2023; Republic of Moldova, 2016; Rumania, 2019, 2022; Serbia, 2016, 2022, 2024; The Netherlands (The Kingdom of Europe), 2017, 2023; The Netherlands (Aruba), 2023; The Netherlands (Curaçao), 2023; Türkiye, 2020; Ukraine, 2018; United Kingdom (Northern Ireland), 2019
Rule 22.2 *The requirements of a nutritious diet*,* including its minimum energy and protein content*,* shall be prescribed in national law*.	Not explicitly referred to in CPT reports.
Rule 22.3 *Food shall be prepared and served hygienically.*	Armenia, 2016; Azerbaijan, 2018, 2018; Bulgaria, 2015; Greece, 2022; Italy, 2017; Lithuania, 2018; Malta, 2016; North Macedonia, 2017, 2024; Republic of Moldova, 2020; Serbia, 2016, 2022; United Kingdom (England and Wales), 2020
Rule 22.4 *There shall be three meals a day with reasonable intervals between them.*	Belgium, 2016, 2018; Bosnia and Herzegovina, 2016; Estonia, 2024; France, 2017; Greece, 2022; Hungary, 2024; Ireland, 2020; Italy, 2017, 2023; Lithuania, 2018, 2023, 2024; Malta, 2016; Montenegro, 2023; North Macedonia, 2017, 2021, 2021, 2024; Portugal, 2018; Republic of Moldova, 2016; Rumania, 2019, 2022; The Netherlands, 2017; The Netherlands (The Kingdom of Europe), 2023
Rule 22.6 *The medical practitioner or a qualified nurse shall order a change in diet for a particular prisoner when it is needed on medical grounds.*	Hungary, 2024; Republic of Moldova, 2016; The Netherlands (Sint Maarten), 2023; Türkiye, 2020

### Inadequacy of food provision

Results are concentrated in Committee concerns and identified violations regarding the provision of adequate and nutritious food in prisons, often in combination with concerns or deficits regarding adequate hygiene, sanitation, access to clean water and or medicines. Particular CAT narratives related to the State obligation to *“guarantee prisoners access to adequate food”* and to address deficits in provision of *“adequate quantities of food and to good quality food”* within the parameters of conditions of detention and the consequences of overcrowding (UN Nelson Mandela Rules 22.1, 42; European Prison Rules 22.1; 22.4). Police stations were also referred to in CAT and Human Rights Committee concerns regarding food shortages and poor conditions of remand detention (Canada [57], Guatemala [58], Namibia [59]– [60], Niger [61], Nigeria [62], Poland [63], Suriname [64]).

Both the Human Rights Committee and the CAT *Concluding Observations* presented grave concern with regard to food rationing and restriction, malnutrition, deaths in custody and hunger strikes (Belize [65], Cuba [66], Democratic Republic of the Congo [67], Madagascar [68], Nicaragua [69]– [70], Syrian Arab Republic [71]). In 2024 in Cyprus, the CPT recommended *“(.) the establishment of a formal written procedure on managing hunger strikes by prisoners.”* [72] A similar recommendation was made regarding Hungary [73] and Romania [74]. 

Some Committees documented issues regarding restricted provision of cutlery (e.g. knives and forks) due to security concerns, insufficient seating arrangements and lack of time available to eat (Azerbaijan [75], Croatia [76]– [77], Greece [78]). For example in 2023 in Croatia it was reported that *“(.) the number of seating places at the table was insufficient*,* obliging prisoners to take turns to eat.”* [76] In Azerbaijan in 2018 it was observed that *“(.) because of the overcrowding prisoners had to hurry when eating (they were taking their meals in 15-minute shifts)”* [75]. The distribution of food in buckets was observed in several countries (Republic of Moldova [79], Serbia [80]). The CPT report from Belgium in 2022 referred to staffing challenges during strikes in the external provision of food to the prison and in the distribution of meals, including hot meals [81]. In an earlier visit in 2016, the CPT had noted *“(.) it is important to take into account the specific nature of deprivation of liberty*,* which places prisoners in a situation of complete dependence on the staff working in the establishment*,* whether in terms of food provision (…).”* [82] Several CPT reports referred to food which was served frozen, cold or lukewarm (e.g. France [83], Belgium [82]).

Whilst detailed recommendations are provided for the State to address, only several CAT reports made explicit recommendations for governments to increase budgetary allocations to support food provision (Bulgaria [84], Burkina Faso [85], Cuba [66], Guatemala [58], Kiribati [86], Togo [87]).

### Poor food Preparation practices, environmental health standards and disease

A small number of CPT reports referred to the requirement for CoE member states to uphold the medical/public health inspection of food standards (UN Nelson Mandela Rule 35.1). Several documented the violation of hygiene standards in food preparation (European Prison Rule 22.3) leading to contaminated food, rodent and disease outbreaks (Azerbaijan [88], Malta [89], Serbia [90]). Unhygienic practices also included eating in cells in close proximity to toilets. For example regarding Ireland, in 2017 the CAT noted concern that *“(.) in-cell sanitation continues to be problematic as 56 persons still have to slop out and 1*,*539 prisoners are required to use toilet facilities in the presence of another inmate*,* in cells where prisoners also have to take their meals.”* [91].

### Reliance on external support for food, corruption and exploitation

Reliance on external supports such as family or well-wishers in providing adequate food in prisons and in police holding cells was observed in several CAT reports (Armenia [92], Niger [61]). For example the 2019 CAT report on Niger commented on the precarious situation of remand prisoners and noted that *“(.) with regard to remand prisoners*,* whose conditions of detention are a matter of particular concern*,* the Committee notes that (.) no food is provided*,* which means that prisoners are entirely dependent on their family members*,* if they have any*,* for food and water.”* [61] Similar was reported in Azerbaijan in 2024 where the CPT noted *“(.) prisoners were expected to (.) using their or their families’ financial resources*,* unless they were destitute and with no one to support them financially (.).”* [93].

Corruption, extortion of people in prison and their relatives by staff, stealing of food by prison staff, inability to complain, and prisoner caste systems, internal cell and gang hierarchies controlling access to food were observed by the CAT (Bangladesh [94], Guatemala [58], Vietnam [95]) and the CPT (Lithuania [96–98], Ukraine [99]). In Guatemala, the CAT reported in 2018 *“(.) the Committee takes note with concern of the persistence of the practice known astalacha*, *whereby bribes are extorted from persons deprived of their liberty in exchange for the avoidance of physical punishment and/or as a condition for the provision of medical treatment*,* food or any other prison benefit (.).”* [58] Women were identified as especially at risk of sexual exploitation in return for food or basic provisions (e.g. the 2024 CAT report on Ecuador [100]).

### Food as punishment and control measure

Some CAT reports included reference to legal permissibility of food restriction as a punishment (UN Nelson Mandela Rule 43.1). For example the 2022 CAT report on Botswana referred to *“(.) the law of the State party continues to allow for the use of reduced diet as a disciplinary measure in prison settings.”* [101] In Kuwait, the 2024 CAT report stated that *“(.) the Committee remains concerned at reports that the Prisons Act still provides for disciplinary measures for prisoner misconduct that constitute violations of the Convention*,* such as (…) the deprivation of certain types of food for a week.”* [102] Other reports indicated grave concern regarding denial or restriction of food as punishment. The CAT report on Vietnam in 2018 indicated concern that *“(.) insufficient quality and quantity of food*,* lack of outdoor physical exercise*,* inadequate health care and severe overcrowding*,* all of which*,* taken together*,* may amount to ill-treatment or even torture*,* and reports that some of these conditions are maintained deliberately as an additional punishment for the inmates.”* [95] The 2016 CPT report on Bosnia Herzegovina reported on the solitary confinement of a prisoner for two days, held in a stress position without access to food or the bathroom [103]. Similar was reported by the CPT during a visit in 2018 to the Czech Republic, where a prisoner was held in a crisis cell naked including when served food [104]. The 2018 CAT report on the Maldives observed the vulnerability of political prisoners and documented *“(.) the frequent use of prolonged solitary confinement*,* in particular against political prisoners; as well as giving them rotten or expired food.”* [105] Similar was reported in the CAT report on Vietnam in 2018 which highlighted the food deprivation experienced by persons sentenced to the death penalty [95]. 

### Vulnerability of special populations

With regard to special populations and considerations for their nutritional and health needs, several Committees (CAT, CEDAW, CRC) reported on the inadequacy of food provision for pregnant/post-delivery/breastfeeding mothers (Bangkok Rule 48.1; 48.3) (Burundi [106], Burkina Faso [107], Central Africa Republic [108], Eritrea [109], Honduras [110], Malawi [111], Niger [112], Tajikistan [113], Türkiye [114], Yemen [115], Zambia [116], Zimbabwe [117]). The 2022 CAT report on Malawi also noted concern regarding poor conditions *“(.) including with regard to (.) food (.)*,* including as regards the specific needs of women*,* pregnant women and mothers with children in detention.”* [118] The CRC identified the impact of food scarcity and inadequate provision impacting on children detained with their mothers in several countries (Angola [119], Lesotho [120], Mauritius [121], Mexico [122]).

Regarding the juvenile justice system, the CRC documented concerns around access to sufficient food regarding juveniles in detention (Argentina [123]– [124], Cambodia [125], Djibouti [126], Guinea [127], Republic of Moldova [128], Rwanda [129], Samoa [130], Seychelles [131] and Vietnam [132]) (Rules for the Protection of Juveniles Deprived of their Liberty Rule 37). Several referred to the inadequacy of food for pregnant juveniles (Bulgaria [133]) and young girls (Republic of Korea [134]). The denial of food by the Israeli authority to Palestinian children in prison was documented by the CRC in 2024 [135]. 

Several CPT reports observed complaints regarding the lack of provision of religiously appropriate food in prisons (Estonia [136], Serbia [137]). The 2021 CPT report on Armenia observed the need for the state to provide appropriate food for foreign national prisoners *“(.) the Committee recommends that steps be taken to ensure that the special dietary requirements of foreign prisoners and prisoners belonging to different religious communities are met (.).”* [138] Regarding individuals with specific medical needs (European Prison Rule 22.6), the CPT Report on the Republic of Moldova referred to the lack of special diets for prisoners with diabetes [139]. In 2020 in Türkiye, the CPT noted that; *“(.) medical staff were unable to give any details about the type of diets available from the kitchen….there was a lack of appetizing*,* low-calorie and low-fat diets as well as of protein supplements for emaciated and cachectic inmates.”* [140].

## Discussion

To date, this is the first global socio-legal assessment of the extent to which minimum standards of care in prisons are upheld as it concerns food provision and quality documented by UN Human Rights Treaty Bodies and European CPT inspections of prisons. Despite international and regional human rights norms and assurances, many States are failing in their duty of care of people in prison extending beyond the prohibition of torture and discrimination and centering on the basic human need for sustenance. In many cases Committee reports included mention of food inadequacy (amount, nutrition) along with deficits in provision of adequate hygiene, sanitation, clean drinking water and space. There is a glaring discrepancy between the detail of scrutiny regarding adequacy of food provision in prisons visited from the respective Treaty Bodies in contrast to the European CPT which provided far greater in-depth assessment of the extent to which European prison standards (particularly European Prison Rule 22) are upheld regarding nutrition, food provision and preparation, environmental health standards, nutrition for special populations and medical oversight of food quality and quantity.

Inspection committees underscored how in resource constrained conditions, food is a commodity, with overcrowding, prisoner caste systems, food deprivation and resource scarcity fueling interpersonal violence, corruption, the stealing of food by staff, transactional sexual activity and reliance on external sources for support (e.g. family). People in prison without family support are extremely vulnerable to food deprivation. This is especially the case in prisons with shared or self-governance structures [141]. Of grave concern are the reports of hunger strikes, food deprivation and official use of food denial as instrument of punishment and control, potentially constituting cruel, inhuman or degrading treatment, or even torture. For special populations of prisoners, a broad range of deficits in nutritional provisions was documented, despite norms and standards highlighting the need for special diets in accordance with age, gender and medical needs, or in accordance with cultural and religious practices [9]. 

There is evidence that the prison food environment, including food supply, quality, nutritional content, food education and culinary skills have the potential to enhance the social connectivity and mental health of the prison community, including through reduction of violence [17, 35, 142–144]. The collated records also reveal progress and promising practices in some countries over time. Many identified good practices are cognizant of the social meaning of food relating to preparation and cooking, autonomy in choosing what to eat and cooking one’s own meals, and eating meals in a communal space. Prison systems which provide opportunities for cooking and sharing food that better reflects familial and cultural identity can improve prison dynamics, relationships, self-esteem and help build life skills for reintegration.[^17, 145^] The CPT visit to the Netherlands in 2016 noted *“(.) prisoners had the possibility of choosing between pre-packed frozen meals or cooking for themselves the food provided by the outside caterer*,* usually collectively. This possibility was welcomed by the inmates as it improved the quality of their meals (.).”* [146] Eating in communal areas was mentioned in several CPT reports (Armenia [147], Bosnia and Herzegovina [148], Denmark [149], Germany [150], Greece [78], Luxembourg [151], Switzerland [152] and the United Kingdom [153]. Other such promising practices included access to kitchenettes and how people in prison are permitted to have their own refrigerators and portable cooking devices in cells (e.g. Albania [154], Armenia [147], Finland [155], Republic of Moldova [156]). In Denmark in 2024, the CPT observed *“(.) the cells to be generally suitably furnished ((.) fridge) (.). There seemed to be no problems with (.) the quality and quantity of the food. Many prisoners (.) could cook their own meals; (…) The delegation was impressed by the initiative of opening supermarket outlets at Nyborg and Enner Mark Prisons*,* where inmates could make their shopping almost in the same manner as people in the outside community (.).”* [149] Prison shops in European prisons also play an important role, despite high prices (e.g. Bulgaria [157]), and in some prisons were replaced by electronic procurement systems (e.g. Albania in 2024 [158]).

European CPT reports also referred to good practices relating to employment and vocational training in prison kitchens and bakeries within the parameters of prison ‘Throughcare’ in preparation for return to the community. Several observed how people in prison are remunerated to work in kitchens and bakeries, preparing food for the prison community and in food distribution (e.g. Albania [154], Andorra [159], Czech Republic [104], Georgia [160], Germany [150], Greece [161], Italy [162]– [163], North Macedonia [164]– [165], the United Kingdom [166]). Other CPT reports documented how prisons provide vocational training relating to food preparation, culinary skills and the service industry to people in prison in preparation for the return to the community (e.g. Albania [167], Austria [168], Belgium [169], Bosnia Herzegovina [170], Estonia [136], Latvia [171]– [172], Liechtenstein [173], Spain [174–176], United Kingdom [166, 177]).

There is a strong evidence base that improved nutrition and integration of food preparation and consumption into prison routines can increase levels of self-efficacy, pro-social identities and resilience, reduce levels of tension, anxiety and depression, and support rehabilitation [17, 145]. Innovative prison food-related interventions include nutritional education, therapeutic horticulture, inclusion of healthy choices in the prison shop and culinary training [35]. There is however also a wealth of evidence that supports that people in prison are exposed to an increased risk of long-term multi-morbidities associated with nutritional deficits including malnutrition, sedentarism and obesity [178]. Nutritional status monitoring of people in prison and appropriate interventions to improve their nutritional status are important particularly concerning communicable and non-communicable disease determinants, immunity levels and co-morbidity [179]. 

More recently, Penal Reform International has reported on the increasing trend to establish environmentally sustainable **‘***green’* prisons, and the rise in implementation of food security and therapeutic initiatives through agricultural training and sustainable food production [1]. Further research on the impact of the prison food environment on health and criminal justice outcomes, and evaluations of prison food provision are warranted to enhance the evidence base in order to inform public and prison health (and nutritional) policy and practice, support increased resourcing and oversight mechanisms, and guide innovative and contextually sustainable solutions for enhanced food security and rehabilitation.

### Strengths and limitations

Strengths of this study lie in its systematic search and analyses of multiple sources of UN Human Rights Treaty Bodies (Human Rights Committee, CAT, CRC, CEDAW) and European CPT inspection reporting on selected prisons worldwide since 2015. The lack of generalizability to the whole prison system of a particular country is a limitation.

## Conclusions

People in prison are wholly reliant on the State to protect and uphold their fundamental human rights whilst detained. Little is known about the scale and effects of food insecurity, food deprivation, undernutrition and related health problems, in prisons worldwide. Understanding the scale of food deprivation and undernutrition in prison, internal risk dynamics including violence and corruption due to food scarcity in prisons, and related health, immunity and disease outcomes is much needed, and can help inform integrated nutrition, health and ‘Throughcare’ programming, oversight mechanisms and public health surveillance spanning prison clinic and public health systems. UN Human Rights Treaty Bodies and Council of Europe Committee inspections must continue to assess food standards and provision in prisons, ensure the denial or restriction of food as punishment is prohibited, and include a focus on those with age-related, religious and medical needs.

## Supplementary Information

Supplementary Material 1.

## Data Availability

Supplemental file of data is uploaded.

## Abbreviations

CAT: Committee against Torture
CPT: Committee for the Prevention of Torture and Inhuman or Degrading Treatment or Punishment
CRC: Committee for the Rights of the Child
CEDAW: Committee on the Elimination of Discrimination against Women
CoE: Council of Europe
UN: United Nations

## Footnotes

1 Prison foodways refers *‘to the behaviors and activities related to the acquisition*,* preparation*,* distribution*,* and consumption of food in prison’*.[48].
